# Concurrent Pediatric Lingual and Submental Dermoid Cysts: Case Report and Literature Review

**DOI:** 10.7759/cureus.42429

**Published:** 2023-07-25

**Authors:** Natasha Gleichmann, Elizabeth Creighton, Austin Zhu, Nicholas Willard, Jeremy Yang, Brian W Herrmann

**Affiliations:** 1 Otolaryngology - Head and Neck Surgery, Children's Hospital Colorado, Aurora, USA; 2 Otolaryngology - Head and Neck Surgery, University of Colorado School of Medicine, Aurora, USA; 3 Pathology, University of Colorado School of Medicine, Aurora, USA; 4 Pathology, Children's Hospital Colorado, Aurora, USA

**Keywords:** paediatric ent, submental, dermoid cysts, sublingual cyst, pediatric case

## Abstract

This pediatric case report describes the novel finding of concurrent submental and lingual dermoid cysts, which to our knowledge, has not been previously reported in the literature. The etiology of cysts involving the tongue, floor of the mouth, and submental neck is varied, representing congenital, inflammatory, and neoplastic sources. Dermoid cysts involving these regions are uncommon and are most frequently reported in the submental, sublingual, and lingual spaces. Presenting symptoms vary with cyst size and position relative to the mylohyoid muscle. MRI is the preferred modality to differentiate dermoid cysts from other etiologies. While interventional techniques have been utilized to treat dermoid cysts in other head and neck locations, surgical excision remains the preferred treatment for those involving oral and floor-of-mouth structures.

## Introduction

Cystic lingual and floor-of-mouth masses in children often present a diagnostic dilemma due to the wide array of potential etiologies, including inflammatory pseudocyst (ranula), infection, lymphatic or vascular malformation, branchial cleft remnant, thyroglossal duct cyst, and dermoid/epidermoid cyst. Although separated from the floor of the mouth by the mylohyoid muscle, many authors include the submental space in discussions of the floor of the mouth due to its shared embryology [1]. Masses involving this region most commonly occupy three anatomic spaces: the submental, sublingual, and midline lingual septum. The midline submental space is demarcated laterally by the anterior bellies of the digastric muscles, superiorly by the mylohyoid muscle, and superficially by the platysma muscle and the superficial cervical fascia. The bilateral sublingual spaces are bounded superiorly by oral cavity mucosa, laterally by the mandible, medially by geniohyoid and genioglossus, and inferiorly by mylohyoid muscles. The lingual septum contains a potential midline space between the genioglossus and geniohyoid muscles extending into the intrinsic tongue musculature. Diagnosing dermoid cysts presenting within the floor of the mouth is complicated by their relative rarity and lack of pathognomonic presenting features. Although 7-8% of dermoid cysts involve the head and neck, only 1.6% are reported on the floor of the mouth [2,3]. Lingual dermoid cysts are exceptionally rare in children, with only 17 reported in the literature [4]. Considered dysembryogenetic lesions, dermoid and epidermoid cysts are thought to derive from entrapped midline endodermal and ectodermal remnants during the fusion of the first and second branchial arches during the third and fourth embryonic weeks [5,6]. A histopathological analysis is used to distinguish between dermoid, epidermoid, and teratoid cysts [7]. While these dysontogenetic cysts can appear during childhood, they are most commonly present between 15 and 35 years of age, with equal frequency in males and females [6,8]. A preceding history of oral trauma noted in a small subset of patients has also suggested a possible inflammatory role in cyst formation [9]. Imaging may not always identify the underlying etiology, requiring physicians to maintain a high index of suspicion for these relatively rare oral cysts. This report describes a case involving the coexistence of lingual and submental dermoid cysts in a pediatric patient, which to our knowledge, has not previously been reported in the literature. A literature review was conducted to provide additional insights to improve the recognition of dermoid cysts involving the lingual and floor-of-mouth regions. 

## Case presentation

**Figure 1:**
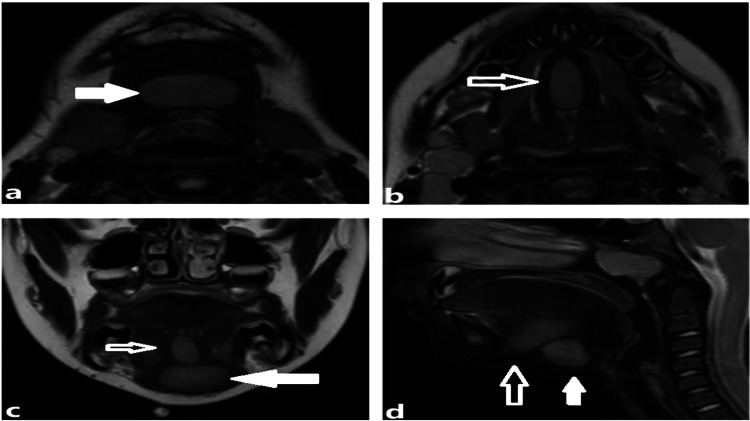
Axial T2 MRI demonstrating (a) submental space and (b) lingual masses. Coronal and sagittal T2 MRI (c & d) demonstrating relative positions of both lesions (submental space mass: solid arrow, lingual mass: clear arrow).

A six-year-old otherwise healthy female was referred to the Pediatric Otolaryngology Clinic to evaluate a non-tender, slowly enlarging submental mass. Although no concerns for dysphagia, voice changes, or stridor were associated with the mass, the patient did report the gradual onset of a fullness in the lower tongue that was noted with swallowing over the past year. Physical examination revealed a midline 2 cm oval mass in the submental neck. The mass moved with swallowing, was non-tender, and was sluggishly mobile to palpation. The floor of the mouth was soft, but the mass was palpable on bimanual palpation. Aside from longstanding mild bilateral reactive cervical lymphadenopathy, the rest of her examination was normal. The lack of any neoplastic stigmata suggested a cyst of congenital or inflammatory origin was the most likely etiology. Initial ultrasound suggested the presence of two masses, prompting a contrasted MRI. Two well-circumscribed ovoid lesions involving the lingual tissue and submental space were identified on MRI (Figure 1). The palpable inferior mass was located within the submental space, anterior to the midline hyoid, measuring 15 x 24 x 17 mm. The second spindle-shaped mass (25 x 8 x 14 mm) was anterior and superior to the submental mass, located within the midline lingual septum, between the geniohyoid and genioglossus muscles. Both nonvascular masses demonstrated similar MRI characteristics (hyperintense on T1, heterogenous on T2), suggesting the masses to be dermoid or epidermoid cysts (Figure 1). 

**Figure 2:**
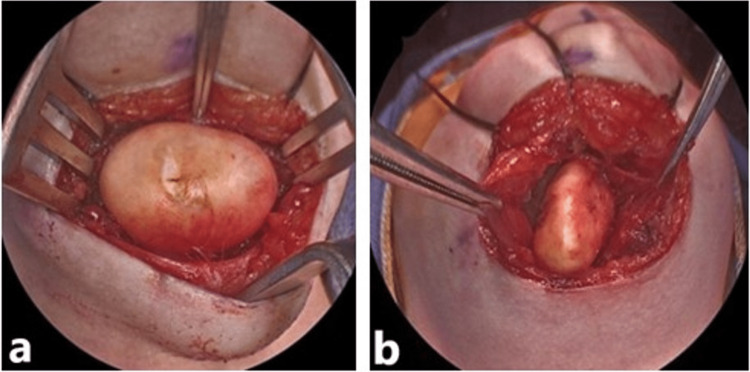
Submental (a) and lingual (b) dermoid cysts.

Surgical excision was performed through a transcervical approach due to the submental location of the inferior cyst. A midline neck incision allowed for the most direct approach to the midline submental mass. The cystic mass was identified and separated from the mylohyoid superiorly and anterior bellies of digastric laterally, with blunt dissection demonstrating the cyst to be well-encapsulated (Figure 2). After removing the inferior cyst, access to the superior cyst was gained by dividing the mylohyoid anteriorly to the mentum to access the geniohyoid and genioglossus muscles. These were separated along their midline raphe until the superior cyst was encountered (Figure 2). Vascular pulsations from lingual arterial branches on either side of the lateral cyst wall were noted. However, the well-circumscribed capsule permitted blunt dissection to facilitate removal while limiting the potential for vascular injury. The superior cyst was excised without violation of the floor of mouth mucosa. Closure of the midline wound proceeded from intrinsic tongue musculature to mylohyoid to submental skin without complications, and an intraoperatively placed drain was removed the next day before discharge.

**Figure 3:**
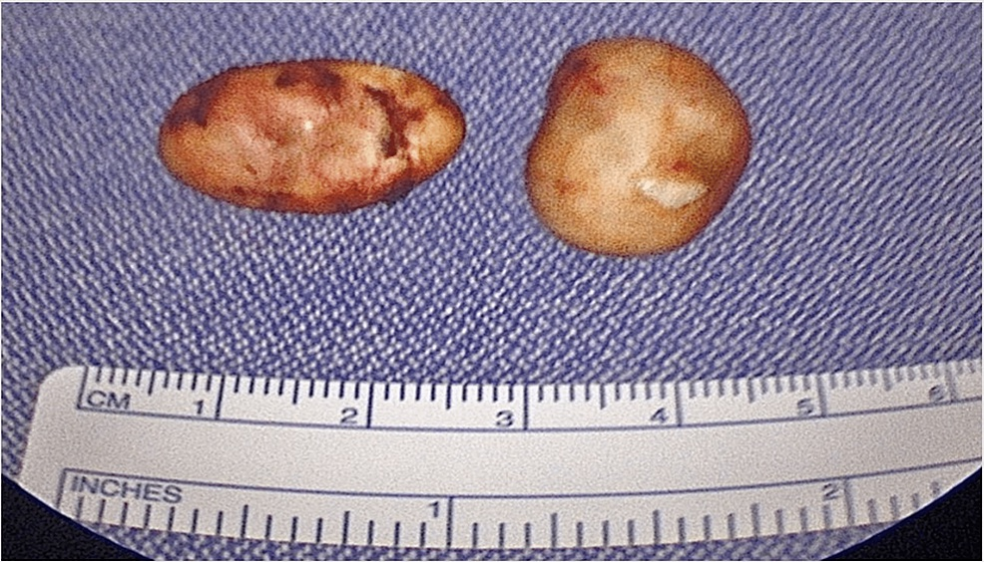
Lingual (l) and submental (r) dermoid cysts.

**Figure 4:**
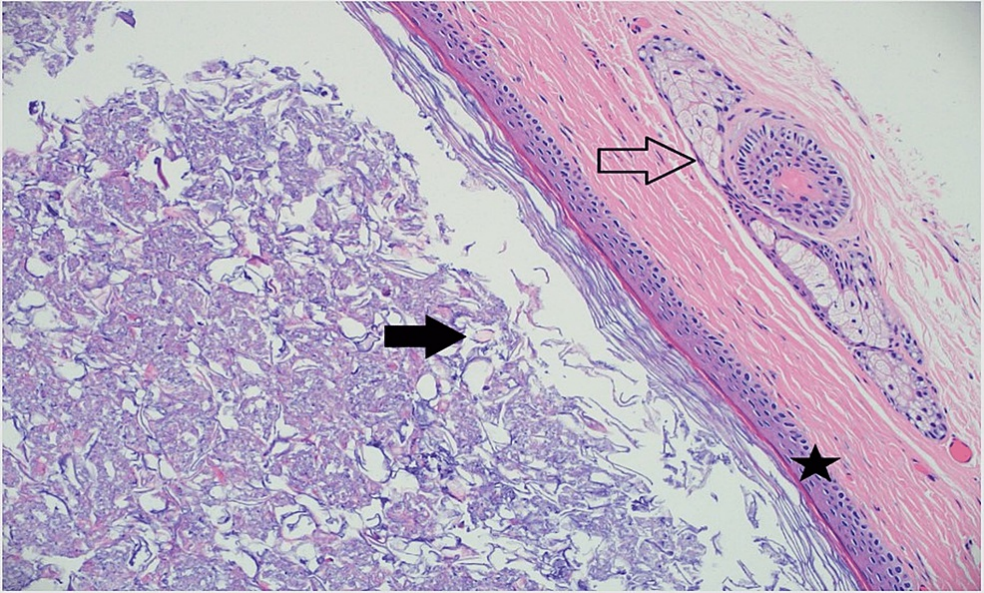
A 20x representative H&E section demonstrates a unilocular cyst lined by stratified squamous epithelium (star). The lumen contains abundant keratin debris and rare hair-like fragments (solid arrow). The cyst wall contains skin adnexal structures such as pilosebaceous units (clear arrow), typical of a dermoid cyst. There is no cytologic atypia or malignancy.

Histopathological examination confirmed both masses to be dermoid cysts. Both masses were grossly described as intact, tan, soft material-filled cysts with a thin, smooth wall cavity exuding caseous tan soft material (Figure 3). Microscopically, both masses showed similar histologic findings, unilocular cystic space lined by stratified squamous epithelium with a preserved granular cell layer and lumens filled with keratinaceous debris, consistent with dermoid cysts (Figure 4). Neither mass was found to have significant inflammation, necrosis, or concern for malignancy. No evidence of recurrence has been noted with follow-up examinations.

## Discussion

Dermoid cyst is often used to collectively refer to three histologic entities: epidermoid, dermoid, and teratoid cysts, as described by Meyer I [7]. An epidermoid cyst is a keratin-filled developmental cyst lined by stratified squamous epithelium lacking adnexal structures, while dermoid cysts are differentiated by the presence of adnexal structures such as sebaceous elements, sweat glands, and hair follicles. Teratoid cysts are rare entities that contain derivatives of all three germ layers, potentially including mesodermal (muscle, vascular tissue, bone) and endodermal (gastrointestinal, bronchogenic tissue) elements. Dermoid cysts within the pediatric oral cavity and adjacent anterior neck spaces share a common embryological origin. They are found with decreasing frequency in the sublingual space, submental space, and lingual tissues [6,10]. Dermoid cysts superior to the mylohyoid muscle are more often associated with respiratory and swallowing concerns than those below the mylohyoid [5]. While sublingual and lingual dermoid cysts are more commonly visible on intraoral examination, submental cysts more often present with external neck swelling. Lingual (or intralingual) dermoid cysts are rare, with only 17 cases reported in the literature [4,10,11]. Most often midline, these are located in the potential space of the lingual septum, a fibrofatty fusion plane between the bilateral lingual masses [12]. Like sublingual space dermoid cysts, these lesions can be associated with speech and swallowing difficulty and also have the potential for upper airway obstruction. As illustrated in this case report, lingual dermoid cysts may be hidden within the tongue musculature, not as obvious on examination as those in the sublingual spaces. Although Edwards PC et al. did report a child developing a midline cervical dermoid cyst 3.5 years after the removal of a lateral lingual cyst, to our knowledge, this case is the first to describe the simultaneous presence of a lingual and submental dermoid cyst in a pediatric patient [10]. Coexisting submental and sublingual dermoid cysts and sublingual dermoid cysts close to bronchogenic and heterotopic gastrointestinal cysts have also been reported [13-15]. As this patient did not experience prior trauma to the oral cavity, the simultaneous slow progression of multiple midline lesions of similar size is most consistent with the enclavement of ectodermal tissue during midline fusion of the bilateral first and second branchial arches during embryogenesis. 

Although consistently described as having a “doughy” or firm consistency on bimanual palpation of the floor of the mouth, accurate diagnosis of these lesions can be difficult based on examination alone [6,16]. A recent pediatric series reported diagnostic accuracy of less than 20% of patients with sublingual dermoid cysts based on examination alone [17]. Pain or rapid expansion is typically not seen unless infection is present [3]. MRI has demonstrated superior differentiation of epidermoid and dermoid cysts from other etiologies than either CT or ultrasound, demonstrating low T1 signal with homogenous hyperintensity on T2 [18]. Our review supports prior reports demonstrating MRI as the preferred imaging modality compared to CT for cystic masses involving the tongue and floor of the mouth [18,19]. Our case is also consistent with Misch E et al., which noted the increased value of imaging when multiple cysts were present [17]. Although fine needle aspiration has been described in diagnosing floor-of-mouth dermoid cysts, this is not routinely employed in the pediatric population [20]. Although no standard treatment algorithm exists for treating lingual and floor-of-mouth dermoid cysts, surgical excision is the treatment of choice for these lesions. Most lingual and sublingual dermoid cysts can be removed with an intraoral approach, while submental dermoid cysts are best removed through an external approach [13,21,22]. In contrast to anterior lingual dermoid cysts amenable to a midline glossectomy approach, the relatively posterior position of our lingual dermoid cyst within the lingual septum made an inferior approach through the midline mylohyoid to access the lingual septum between the geniohyoid and genioglossus muscles the preferred approach. This decision was also influenced by the easy exposure of the mylohyoid immediately superior to the resected submental dermoid cyst. Complete surgical excision is associated with excellent long-term prognosis with minimal recurrence risk [6,13]. 

## Conclusions

Dermoid cysts involving the tongue and floor of the mouth are uncommon in pediatric patients, with lingual involvement being rare. To our knowledge, this report provides the first description of simultaneous lingual and submental dermoid cysts in a pediatric patient. Difficulties with swallowing or respiration were more common with lingual and sublingual dermoid cysts, while submental lesions more commonly presented as a neck mass. A literature review suggested that intraoral and external surgical approaches were preferred for dermoid cysts above and below the mylohyoid. Surgical planning should be individualized to minimize morbidity, particularly in the presence of multiple lesions.
